# Plasmacytoma as a cause of small bowel obstruction in a virgin abdomen in a patient with multiple myeloma: a case report

**DOI:** 10.1186/s13256-019-2068-x

**Published:** 2019-05-17

**Authors:** Aubrey A. Mwinyogle, Astha Bhatt, Katarina Kapisoda, Justin Somerville, Steven C. Cunningham

**Affiliations:** 1 Department of Surgery, St Agnes Hospital, Baltimore, MD USA; 2 Colorectal Surgery, St Agnes Hospital, Baltimore, MD USA; 3 Pancreatic and Hepatobiliary Surgery, St Agnes Hospital, 900 Caton Avenue, MB 207, Baltimore, MD 21229 USA

**Keywords:** Multiple myeloma (MM), Plasmacytoma, Extramedullary plasmacytoma (EMP), Small bowel obstruction (SBO), Intussusception

## Abstract

**Background:**

Multiple myeloma is a hematological malignancy that classically results in an abnormal clonal proliferation of plasma cells in the bone marrow. Extramedullary disease in the setting of multiple myeloma, referred to as secondary extramedullary plasmacytoma, is found in 7–17% of cases of multiple myeloma at the time of diagnosis and can involve any organ system. Small bowel obstruction is a rare but important gastrointestinal manifestation of multiple myeloma that should be considered in patients with multiple myeloma who present with concerning abdominal symptoms.

**Case presentation:**

We present the case of a 52-year-old African-American man with a history of deep venous thrombosis (he is on anticoagulation) and pathologic fracture secondary to multiple myeloma diagnosed 4 months prior to our encounter. He presented with abdominal pain, constipation, nausea, and vomiting. An abdominal X-ray showed distended bowel loops concerning for bowel obstruction and a contrast-enhanced computed tomography scan of his abdomen and pelvis showed a 5.4 cm soft tissue mass involving a loop of distal ileum. He underwent laparoscopic exploration of his abdomen with small bowel resection and primary anastomosis for a small intussusception. He had an uneventful postoperative course and was discharged on postoperative day 6.

**Conclusions:**

Multiple myeloma has myriad presentations. Gastrointestinal involvement, although rare, can manifest as small bowel obstruction for which early recognition and appropriate surgical management are key to improving outcome. Intussusception is the most common mechanism of obstruction from extramedullary plasmacytoma causing small bowel obstruction and this has been seen in five of six case reports, including this case. It is important to recognize and consider the risks of immunosuppression, venous thromboembolism, and malnutrition in the surgical management of gastrointestinal complications of multiple myeloma.

## Background

Multiple myeloma (MM) represents 1–2% of all cancers in the USA with an annual incidence of 4.3 per 100,000 [1, 2]. Extramedullary involvement of MM, also known as extramedullary plasmacytoma (EMP), can present at the time of initial of diagnosis or as relapse. EMP has been demonstrated to be associated with decreased overall survival [3, 4]. Furthermore, survival depends in part on the site of extramedullary disease: soft-tissue extramedullary disease having significantly worse overall survival compared to bone-related extramedullary disease [5].

Gastrointestinal (GI) involvement by EMP in MM is rare. In a study by Giampaolo *et al.*, only 24 of 2584 patients with MM (0.9%) were found to have extramedullary disease affecting the GI tract [6]. Given that involvement of the GI tract, however, can be asymptomatic and discovered incidentally on imaging or autopsy, the incidence is probably higher. In fact, this is just what was found in an autopsy study that detected extramedullary involvement in as high as 65% of 57 consecutively autopsied cases of MM [7].

Data on the relevance and role of surgery in GI involvement by MM remain mostly limited to case reports. However, with improvements in therapy and overall survival in MM, coupled with more sensitive imaging, GI involvement and the need for surgical input or intervention will become relatively more common and relevant to the general surgeon [3, 8]. It is thus important for surgeons to be familiar with MM, including understanding the role of immunosuppression due to recent chemotherapy or steroid use and other risk factors such as venous thromboembolism and malnutrition, in the surgical decision making for patients with MM requiring surgical input [9]. This case study adds to the rare but increasingly relevant literature of small bowel obstruction (SBO) from EMP in a patient with MM requiring surgical intervention.

## Case presentation

Our patient is a 52-year-old African-American man with no prior abdominal surgeries and a past medical history of MM and venous thromboembolism who presented with a 6-day history of nausea, vomiting, and abdominal pain. He had not passed flatus for 24 hours prior to presentation. He did not have fever, chills, or malaise. He was seen by his oncologist and an abdominal X-ray was done; the abdominal X-ray was concerning for SBO for which reason he was subsequently admitted and general surgery consulted.

He had been diagnosed as having MM 4 months prior, after sustaining a pathologic left humerus fracture and was on chemotherapy as well as radiation therapy to the affected humerus. He had completed his second cycle of chemotherapy 10 days prior to presenting with signs of SBO. His chemotherapy regimen included bortezomib, lenalidomide, and dexamethasone.

His MM was diagnosed with a bone marrow biopsy that showed 25% clonal plasma cells and Kappa light chain restricted. Serum K/L was 222 and fluorescence *in situ* hybridization (FISH) myeloma cytogenetic analysis detected a 17p13 deletion in 30% of cells and a t(14:16) re-arrangement was detected in 5.7% of cells. These cytogenetic changes are both identified as high-risk features in the Mayo Stratification of Myeloma and Risk-Adapted Therapy (mSMART) molecular risk classification system. He had stage II disease by the International Staging System (ISS) of MM, with serum beta-2 microglobulin of 4.7 mg/L and serum albumin of 4.5 g/dl.

His other medical conditions include hypertension, obesity, sleep apnea, vitamin D deficiency, and pulmonary embolism diagnosed 1 month after his diagnosis of MM, for which he was on therapeutic enoxaparin. He had no prior abdominal operation. His family history is significant for breast cancer in his sister.

**Fig. 1:**
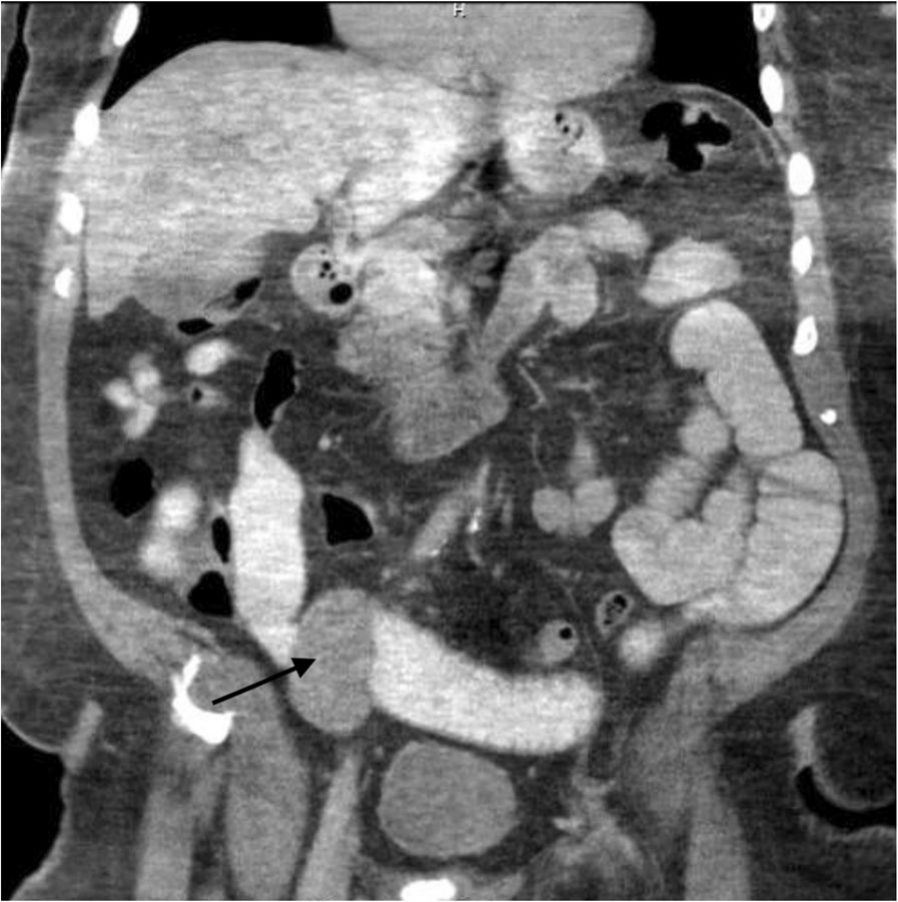
Coronal section of a computed tomography scan of the abdomen showing an area of soft tissue mass (*arrow*) in the small bowel in the right lower quadrant

His vital signs were normal and an abdominal examination revealed tenderness in the right lower quadrant with mild guarding. A contrast-enhanced computed tomography (CT) scan of his abdomen showed a 5.4 cm soft tissue mass involving a loop of distal ileum causing dilation of proximal ileum (Fig. 1). There was also a fluid collection with layering contrast and air in the right peritoneum consistent with a bowel perforation.

He was taken to the operating room for exploration and underwent a laparoscopic small bowel resection with primary anastomosis. A small intussusception was noted intraoperatively. He had an uneventful postoperative course and was discharged on postoperative day 6. A small-bowel mass at the intussusception was confirmed as plasmacytomas on pathology.

He was seen in the surgical clinic 2 weeks postoperatively, doing well. His midline surgical scar was healing well, without any signs of infection. He continued to follow up with his oncologist and his chemotherapy regimen was switched to daratumumab, pomalidomide, and dexamethasone because of his progressive disease (intestinal EMP) despite his initial chemotherapy regimen. He was referred to a tertiary facility because of the aggressive nature of his disease. He developed multiple new skin masses and, subsequently, weakness and sensory changes in bilateral lower extremity with magnetic resonance imaging (MRI) of his spine demonstrating spinal cord compression from T4–T6 level secondary to epidural extension of MM. Despite further aggressive chemotherapy regimen at tertiary facility, including bortezomib, dexamethasone, thalidomide, cisplatin, adriamycin, and cyclophosphamide, he developed worsening and widespread disease with metabolically active masses on positron emission tomography/CT involving right orbit, lungs, liver, spleen, bilateral adrenal glands, and multiple lymph nodes including mediastinal nodes. His clinical and functional status progressively declined, and he died 11 months following his initial diagnosis of MM.

## Discussion

MM is an abnormal clonal proliferation of plasma cells producing a monoclonal immunoglobulin and accounts for 17% of hematological malignancies [1]. Classically, these clonal plasma cells, through a complex signaling pathway involving stromal cell-derived factor 1, chemokine receptor CXCR4, lymphocyte function-associated antigen 1 (LFA-1), very late antigen-4/5 (VLA 4/5), and matrix metalloproteinases (MMPs) 2–9, proliferate in bone marrow resulting in symptoms related to bone destruction and hematopoietic disruption [10]. Extramedullary disease in the setting of MM occurs in 7–17% of cases at the time of MM diagnosis and 6–20% during the course of disease progression [3, 11, 12]. When EMP occurs in the absence of evidence of a plasma cell neoplasm involving the bone marrow, it is referred to as primary EMP.

Small bowel involvement in plasmacytoma secondary to a known plasma cell myeloma (PCM) is quite rare: in a review of the only 61 cases of EMP of the small intestine reported in the literature as of 2012, Lopes da Silva found that only five of these already rare 61 cases were secondary to another, known PCM [13]. However, due to more effective novel chemotherapeutic options the overall survival from MM is expected to increase with an expectant rise in uncommon presentations [14].

GI manifestations depend on the organ involved but generally result from direct organ invasion leading to perforation in cases of hollow viscus involvement, mechanical mass effects leading to obstruction, and, rarely, development of ascites [6].

Although more recent data have suggested that adhesions remain the primary cause of SBO even in the virgin abdomen [15, 16], this case serves as a reminder that neoplasms may of course also cause SBO in virgin abdomens.

To the best of our knowledge, from a search of the English literature using PubMed, Embase, and Google Scholar, only five cases of SBO from plasmacytoma secondary to MM have been reported [17–21]. Among these reported cases in the literature, intussusception was noted in four out of the five cases (80%). In fact, given that there was a small intussusception in our case seen intraoperatively, the incidence rises to 83%: five out of six cases, including ours.

Given an increasing overall survival from MM, the relevance of uncommon presentations of MM, such as SBO, to the general surgeon has become greater than before. This case highlights plasmacytoma as a differential diagnosis for SBO especially in the population of patients with MM with a virgin abdomen who present with concerning abdominal symptoms.

## Conclusion

MM is a hematologic malignancy with myriad presentations that can involve multiple systems including the small bowel where EMP can lead to bowel obstruction and perforation. Small-bowel plasmacytomas appear to have a high likelihood of causing intussusception as noticed intraoperatively in five out of six case reports of secondary plasmacytoma causing SBO, including this case.

Although adhesions remain the most common cause of SBO in the virgin abdomen, the differential of plasmacytoma should be an important consideration in the population of patients with MM especially those without prior abdominal surgery.

Various factors such as the risk of immunosuppression due to MM itself and chemotherapy/steroid use, venous thromboembolism, and malnutrition need to be considered in the planning of surgical intervention (preoperatively and postoperatively) in patients with MM with GI complications.

Surgical experience and the role of surgery with EMP involving the GI system remains limited with most reports in the literature being case reports; more research and case reports are needed to help in guiding the optimal assessment, prognostic evaluation, and decision making for surgical intervention.

## Abbreviations

CT: Computed tomography
EMP: Extramedullary plasmacytoma
FISH: Fluorescence *in situ* hybridization
GI: Gastrointestinal
ISS: International Staging System
LFA-1: Lymphocyte function-associated antigen 1
MM: Multiple myeloma
MMPs: Matrix metalloproteinases
MRI: Magnetic resonance imaging
mSMART: Mayo Stratification of Myeloma and Risk-Adapted Therapy
PCM: Plasma cell myeloma
SBO: Small bowel obstruction
VLA-4/5: Very late antigen-4/5
